# The association between sleep duration and muscle sympathetic nerve activity

**DOI:** 10.1007/s10286-023-00965-7

**Published:** 2023-08-06

**Authors:** Bryan W. S. Tai, Tye Dawood, Vaughan G. Macefield, Stephanie R. Yiallourou

**Affiliations:** 1 Human Autonomic Neurophysiology Lab, Baker Heart and Diabetes Institute, Melbourne, Australia; 2 Department of Neuroscience, Central Clinical School, Monash University, Melbourne, Australia; 3 Turner Institute for Brain and Mental Health, Monash University, Melbourne, Australia

**Keywords:** Sympathetic activity, Cardiovascular disease, Hypertension, Short sleep

## Abstract

**Purpose:**

Sleep duration is associated with risk of hypertension and cardiovascular diseases. It is thought that shorter sleep increases sympathetic activity. However, most studies are based on acute experimental sleep deprivation that have produced conflicting results. Furthermore, there are limited data available on habitual sleep duration and gold-standard measures of sympathetic activation. Hence, this study aimed to assess the association between habitual sleep duration and muscle sympathetic nerve activity.

**Methods:**

Twenty-four participants aged ≥ 18 years were included in the study. Sleep was assessed using at-home 7-day/night actigraphy (ActiGraph™ GT3X-BT) and sleep questionnaires (Pittsburgh Sleep Quality Index and Epworth Sleepiness Scale). Microelectrode recordings of muscle sympathetic nerve activity were obtained from the common peroneal nerve. Participants were categorised into shorter or longer sleep duration groups using a median split of self-report and actigraphy sleep measures.

**Results:**

Compared to longer sleepers, shorter sleepers averaged 99 ± 40 min and 82 ± 40 min less sleep per night as assessed by self-report and objective measures, respectively. There were no differences in age (38 ± 18 vs 39 ± 21 years), sex (5 male, 7 female vs 6 male, 6 female), or body mass index (23 ± 3 vs 22 ± 3 kg/m^2^) between shorter and longer sleepers. Expressed as burst frequency, muscle sympathetic nerve activity was higher in shorter versus longer sleepers for both self-report (39.4 ± 12.9 vs 28.4 ± 8.5 bursts/min, *p* = 0.019) and objective (37.9 ± 12.4 vs 28.1 ± 8.8 bursts/min, *p* = 0.036) sleep duration.

**Conclusions:**

Shorter sleep duration assessed in a home setting was associated with higher muscle sympathetic nerve activity. Sympathetic overactivity may underlie the association between short sleep and hypertension.

**Supplementary Information:**

The online version contains supplementary material available at 10.1007/s10286-023-00965-7.

## Introduction

Sleep disorders such as obstructive sleep apnoea (OSA) and insomnia have been associated with adverse cardiovascular outcomes [1]. While many studies have focused on sleep disorders, there is growing evidence that sleep duration, per se, also impacts cardiovascular health. Shorter sleep duration, in the absence of a sleep disorder, can be caused by lifestyle factors including long work hours and late-night use of electronic devices [2]. Epidemiological studies have previously identified an independent association between sleep duration and hypertension, cardiovascular morbidity, and all-cause mortality [3, 4]. In middle-aged adults, each hour of sleep reduction was associated with a 37% increase in the odds of incident hypertension after 5 years [5]. Furthermore, a U-shaped relationship was identified between sleep duration and blood pressure (BP), with both shorter and longer sleep duration associated with a higher risk of elevated BP [6].

Following either sleep restriction or total sleep deprivation, elevated BP, heart rate (HR), cortisol production, noradrenaline urinary excretion, and low-frequency HR variability occur [7–11]. Taken together, these findings suggest elevated sympathetic activity; however, these studies have relied on indirect measures of sympathetic activity. The gold standard for the measurement of sympathetic activity is microneurography, in which sympathetic nerve activity is recorded directly from postganglionic axons in peripheral nerves. Studies that have utilised measures of muscle sympathetic nerve activity (MSNA) show that full-night sleep deprivation does not alter (or even reduces) sympathetic activity, affecting the set-point of the baroreflex function curve [12, 13]. Furthermore, most of these studies are experimental in design, are acute in nature, and have been performed in a laboratory environment which, while providing vital information, may not be reflective of real-world conditions. Accordingly, there are no studies on whether natural sleep patterns produce similar effects on MSNA to those seen in sleep deprivation/restriction studies.

Measures of habitual sleep duration can be achieved via both subjective (self-report) and objective (actigraphy monitoring) assessments. Actigraphy monitoring is a well-established, validated measure of objective sleep patterning that can be applied over multiple nights in the home setting [14]. Subjective measures of sleep provide information on self-perceived, habitual sleep patterning and are widely used in epidemiological cohort studies due to ease of assessment. Though subjective measures may over- or underestimate sleep duration, self-report sleep duration correlates with objective measures, and taken together, both measures can provide key information on sleep patterning [15].

In this study, we employed gold-standard microneurography to assess MSNA as well as sleep questionnaires and 7-day/night actigraphy monitoring to provide an unobtrusive, objective measure of sleep duration in the home setting. We aimed to (1) assess habitual sleep duration and quality using objective and subjective measures in adults, (2) determine the association between sleep duration and quality with MSNA, and (3) compare individuals with shorter versus longer sleep duration. We hypothesised that sleep duration would be correlated with MSNA and that MSNA would be higher in individuals with shorter versus longer sleep duration.

## Methods

### Study design

The study was approved by the Western Sydney University Human Research Ethics Committee (H11462) and carried out in the Human Autonomic Neurophysiology Lab at the Baker Heart and Diabetes Institute, Melbourne. Written informed consent was obtained from all participants prior to inclusion in the study. Procedures were performed in line with institutional and ethical guidelines, endorsed by the governance of the Baker Heart and Diabetes Institute.

### Participants

This study formed a sub-study of a larger-scale project investigating neural regulation of BP (H11462) where participants underwent MSNA recordings in addition to functional magnetic resonance imaging. Participants who were recruited to this study were invited to take part in an additional assessment of their sleep patterning via subjective (questionnaires) and objective measures (actigraphy). In total, 24 adults (21–82 years; 11 male, 13 female) were recruited for the sleep study.

We performed targeted recruitment of individuals with a range of sleep durations. One important detail of our recruitment and screening strategy was a face-face discussion with participants surrounding inadequate sleep driven by lifestyle behaviours (such as social, leisure, or work-related activities), and that the study’s focus was to investigate sleep duration that does not occur as a consequence of a sleep disorder. Participants were deemed ineligible if they self-reported prior diagnosis of a sleep disorder, snored, or had other exclusion criteria including witnessed apnoea, or problems with initiating or maintaining sleep more than three nights/week for longer than one month. Exclusion criteria further included participants who reported prior clinical diagnosis of cardiovascular disease (CVD), known history of respiratory disturbances, smoking, obesity [body mass index (BMI) > 30], diabetes, hypnotic or stimulant medications, or shift work.

### Procedures

 Once screened, participants underwent microneurography in the laboratory. To assess sleep quantity and quality, participants completed the sleep questionnaires and underwent 7-day/night actigraphy. Detailed procedures for sleep questionnaires, actigraphy, microneurography, and recording analyses are provided in supplemental methods.

### Sleep questionnaires

To assess subjective sleep duration and quality, participants completed the Pittsburgh Sleep Quality Index (PSQI), with a global score of > 5 defined as clinically significant poor sleep quality [16]. The Epworth Sleepiness Scale (ESS) was utilised to assess daytime sleepiness, with scores > 10 considered clinically significant [17].

### Actigraphy

Objective sleep was assessed using 24-h, 7-day/night actigraphy (ActiGraph™ GT3X-BT, ActiGraph, LLC, Pensacola, FL, USA). The device was fitted on participants’ hips along their mid-axillary line. Hip placement was chosen, as this study was part of a larger-scale study which was interested in physical activity data throughout the day as well as nighttime sleep. Hip actigraphy is considered more reliable for physical activity measures [18], albeit less reliable for sleep assessment, than wrist actigraphy [19]. Hip-worn accelerometery, however, has shown stronger correlations with gold-standard polysomnography-derived total sleep duration over wrist-worn actigraphy [19]. Sleep diaries were provided for logging in-bed and out-of-bed times and periods of non-wear. Data were analysed by the same trained researcher with specialised software (ActiLife 6.8.1, ActiGraph, LLC) in 60-s epochs, using a standardised protocol. Activity data and wear times were manually inspected and validated against diary entries, followed by determination of sleep and wake time based on activity using the validated Cole–Kripke algorithm [19].

Days with missing activity or recordings comprising less than four days were excluded. Total sleep time (TST, min), time in bed (TIB, min—derived from in- and out-of-bed time), and sleep efficiency (SE%, a measure of sleep quality—derived by TST divided by TIB) were averaged across the 7 nights of the recording.

###  Microneurography and physiological parameters

Microneurography was performed via previously established protocols [20] (see supplemental methods for detailed protocol). Briefly, MSNA was recorded from muscle fascicles of the common peroneal nerve via a tungsten microelectrode percutaneously inserted into the nerve. Neural activity was amplified (×20,000), filtered (0.3–5.0 kHz) (Neuro Amp EX, ADInstruments, Sydney, Australia), and sampled at 10 kHz. To measure respiration and electrocardiography, a respiratory belt transducer (ADInstruments, Sydney, Australia) and three-lead electrocardiogram (ECG) (Bio Amp, PowerLab, ADInstruments, Sydney, Australia) were used, respectively. Continuous BP was recorded at rest using an appropriately sized, non-invasive photoplethysmographic cuff placed around the left ring or middle finger (NOVA, Finapres Medical Systems, Enschede, Netherlands). Continuous BP measures were calibrated against an oscillometric brachial BP measurement obtained from the non-monitored (right) arm using an upper arm cuff. The height correction unit was zeroed and implemented according to the manufacturer’s specifications. A 15-min recording was conducted in each participant to obtain a 5-min artifact-free recording to be used for calculating baseline parameters. All physiological signals were stored on a computer via a data acquisition and analysis system (PowerLab™ 16:35 hardware and LabChart™ software for Macintosh, v7.1.2.5 software; ADInstruments).

Data were analysed offline using LabChart 7 according to previously published protocols [20]. The analytical methods are described in detail in supplemental methods. Briefly, MSNA bursts were quantified according to burst frequency (BF, bursts/min) and burst incidence (BI, bursts/100 heartbeats). BP and HR were calculated via peak detection of both BP and ECG signals, respectively. Beat-to-beat measures of systolic blood pressure (SBP, mmHg), diastolic blood pressure (DBP, mmHg), mean blood pressure (MBP, mmHg), and HR (beats/min) were obtained and averaged across 10–15 min of baseline recordings for each participant. MBP was calculated using the following equation: MBP = DBP + 1/3(SBP − DBP) [21]. Individuals were classified as hypertensive according to SBP/DBP ≥ 140/90 mmHg or prescribed hypertensive medication [22].

### Statistical analysis

Statistical analyses were conducted using GraphPad Prism (version 9.2.0 for macOS, GraphPad Software, San Diego, CA, USA) and IBM SPSS Statistics version 26.0 (IBM Corp., Armonk, NY, USA). Demographic and clinical characteristics were tested for normality with the Shapiro–Wilk test. Continuous variables were presented as mean ± standard deviation (SD) and categorical variables were expressed as percentages.

Firstly, we compared subjective and objective measures of sleep duration using Student’s *t* test and correlation analysis. Secondly, correlation analysis (Pearson’s *R*^2^ or Spearman’s *R* depending on normality) was utilised to assess the association between TST (assessed by self-report and actigraphy measures) and MSNA (BF and BI). Correlations were also performed between age and MSNA, BMI and MSNA, and BP (SBP, DBP, and MBP) and MSNA. To adjust for potential confounders of age and sex, a backward multiple regression analysis was performed which included TST, age, and sex as independent variables and BF and BI as dependent outcome variables.

Finally, to compare individuals with short versus longer sleep duration, participants were stratified into two groups via a median split of subjective and objective TST. A median split was chosen (1) because a cut-off for short sleep duration in the context of MSNA as an outcome is unknown and (2) to ensure relatively equal groups and sufficient numbers within groups. Group differences were compared using an independent Student *t* test or a Mann–Whitney *U* test for parametric and non-parametric data, respectively, for continuous variables. Categorical data were compared using *χ*^2^ analysis. Sensitivity analysis was also performed (1) to exclude individuals with hypertension, as hypertension is associated with elevated sympathetic activity [23], and (2) to exclude participants with high risk of sleep disorders, with an ESS > 10 and PSQI > 5. A *p* value of < 0.05 was considered statistically significant.

### Sample size

Based on published data, a minimum sample size of *n* = 24 was required to identify a clinically meaningful difference between groups of 12 bursts/100 heartbeats with a standard deviation of 10 bursts/100 heartbeats (*α* = 0.05) with a power of 0.8 [24].

## Results

As shown in Table 1, for the whole group, the mean age was 38.6 ± 19.3 years, mean BMI was in the normal range, and sex proportions were not different. Six participants were classified as hypertensive. One participant reported previous stroke unrelated to CVD. Mean self-report and objective TST ranged between 7 and 8 h, and PSQI and ESS scores were within normal ranges (i.e., < 5 for PSQI and < 10 for ESS).

**Table 1:** Participant characteristics

Participant characteristics	
No.	24
Sex	
Male, *n* (%)	11 (46%)
Female, *n* (%)	13 (54%)
Age (years)	38.6 ± 19.3
BMI (kg/m^2^)	22.8 ± 2.9
Hypertension, *n* (%)	6 (25%)
Subjective sleep measures	
Self-report TIB (min)	470.9 ± 54.9
Self-report TST (min)	431.3 ± 59.1
PSQI (score)	4.9 ± 3.0
PSQI > 5, *n* (%)	6 (25%)
ESS (score)	6.2 ± 4.1
ESS > 10, *n* (%)	2 (8%)
Objective sleep measures	
Objective TIB (min)	473.2 ± 49.9
Objective TST (min)	464.3 ± 52.0
SE (%)	97.5 ± 1.7
Haemodynamic measures	
SBP (mmHg)	124.8 ± 23.2
DBP (mmHg)	74.0 ± 9.6
MBP (mmHg)	90.7 ± 15.0
HR (beats/min)	68.2 ± 10.2

Data presented as mean ± standard deviation unless otherwise specified

*BMI* body mass index, *TIB* time in bed, *TST* total sleep time, *PSQI* Pittsburgh Sleep Quality Index, *ESS* Epworth Sleepiness Scale, *SE* sleep efficiency, *SBP* systolic blood pressure, *DBP* diastolic blood pressure, *MBP* mean blood pressure, *HR* heart rate

### Subjective versus objective measures

TST recorded by self-report was lower when compared to objective measures (431.3 ± 59.1 min vs 464.3 ± 52.0 min, *p* = 0.046). However, correlation analyses indicated a positive relationship between both measures (*R*^2^ = 0.66, *p* < 0.0001; Figure S1).

### Regression analyses

**Fig. 1:**
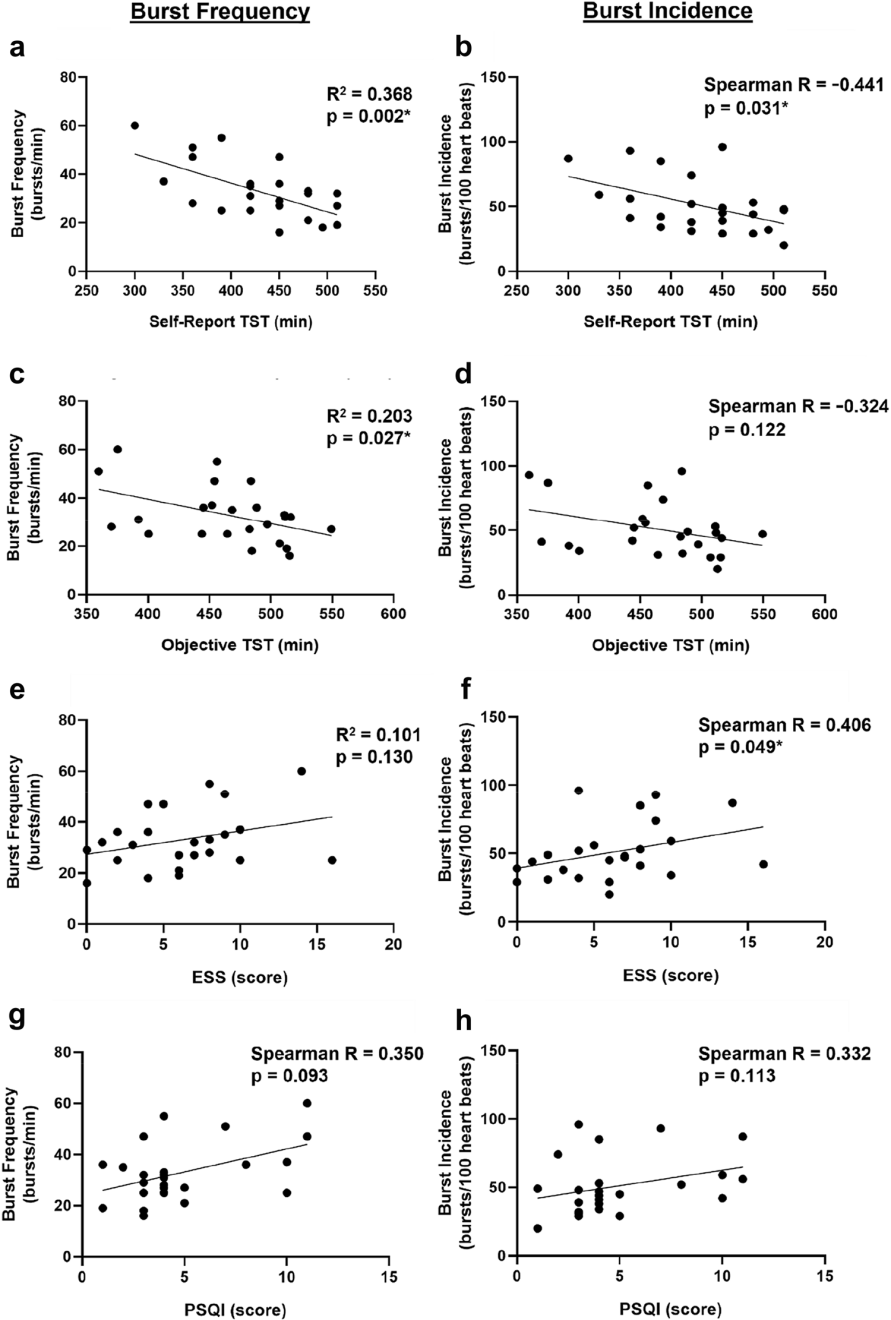
Correlation between sleep duration and MSNA. Correlations between (**a** and **b**) self-report total sleep time (TST, min), (**c** and **d**) objective total sleep time (TST, min), (**e** and **f**) Epworth Sleepiness Scale (ESS, score), and (**g** and **h**) Pittsburgh Sleep Quality Index (PSQI, score) with burst frequency (bursts/min) and burst incidence (bursts/100 heartbeats) (*n* = 24) *p < 0.05

Shorter self-report TST was associated with higher BF (*R*^2^ = 0.37, *p* = 0.002; Fig. 1) and BI (Spearman *R* = −0.44, *p* = 0.031), while shorter objective TST was positively associated with BF (*R*^2^ = 0.20, *p* = 0.027), but not BI. A higher ESS score was positively associated with higher BI (Spearman *R* = 0.406, *p* = 0.049; Fig. 1F), but not BF. No associations were found between PSQI or SE and MSNA. Older age was associated with higher BF (*R*^2^ = 0.36, *p* = 0.002; Figure S4) and BI (Spearman *R* = 0.70, *p* = 0.0001), while there was no association with BMI. BP measures (SBP, DBP, and MBP) were not associated with MSNA. HR was not associated with BF, but higher HR was associated with lower BI (Spearman *R* = −0.543, *p* = 0.006), which is expected given that HR is used to calculate BI.

Using multiple regression, following adjustment for age and sex, shorter self-report TST was associated with higher BF (*β* = −0.11, *p* < 0.001) and BI (*β* = −0.15, *p* = 0.01). Similarly, shorter objective TST was associated with higher BF (*β* = −0.09, *p* = 0.01), but not BI (*β* = −0.13, *p* = 0.051). Overall, older age was associated with higher levels of MSNA measures (*p* < 0.05, for all models).

### Shorter versus longer sleep duration

The median value for self-report TST was 7.5 h and objective TST was 7.93 h. The median values for self-report and objective TST were used to classify participants into shorter (self-report *n* = 10, objective *n* = 12) and longer sleepers (self-report *n* = 14, objective *n* = 12), respectively. As summarised in Table 2, age, BMI, and sex proportions were not significantly different between shorter and longer sleepers. The shorter sleep duration group spent 73–74 min less time in bed and slept approximately 1.5 h less (−99 min TST for self-report, −82 min TST for objective sleep) than longer sleepers. Those with shorter sleep duration had higher ESS and PSQI scores than longer sleepers; however, objective SE% was not different between groups. For BP, four participants had missing BP data due to technical difficulties. Of those with BP data, self-reported shorter sleepers had significantly lower mean DBP and MBP compared to the longer sleep duration group, but no differences in mean SBP or HR. In contrast, for objective TST, no differences were found for any BP or HR measures. The shorter sleep duration group had two individuals with hypertension and the longer sleep duration group had four.

**Table 2:** Group differences for participant characteristics, sleep, and haemodynamics

	Shorter sleep duration	Longer sleep duration	*p*
Self-report TST—grouped by median split of self-report TST^a^			
Participant characteristics			
*n*	10	14	
Sex			
Male, *n* (%)	3 (30%)	8 (57%)	0.188
Female, *n* (%)	7 (70%)	6 (43%)	
Age (years)	38.0 ± 20.3	39.0 ± 19.2	0.655
BMI (kg/m^2^)	23.1 ± 2.7	22.6 ± 3.1	0.661
Subjective sleep measures			
Self-report TIB (min)	427.5 ± 49.6	501.9 ± 33.8	0.0002
Self-report TST (min)	373.5 ± 40.3	472.5 ± 25.5	< 0.0001
PSQI (score)	6.7 ± 3.5	3.6 ± 1.7	0.019
PSQI > 5, *n* (%)	5 (50%)	1 (7%)	0.017
ESS (score)	9.2 ± 3.8	4.1 ± 2.7	0.008
ESS > 10, *n* (%)	2 (20%)	0 (0%)	0.081
Haemodynamic measures			
*N*	9	11	
SBP (mmHg)	120.0 ± 14.8	135.2 ± 27.0	0.091
DBP (mmHg)	69.3 ± 9.0	77.7 ± 8.8	0.049
MBP (mmHg)	82.9 ± 14.7	97.0 ± 12.6	0.032
Heart rate			
*N*	10	14	
HR (beats/min)	67.6 ± 8.4	68.6 ± 11.7	0.812
Objective TST—grouped by median split of objective TST			
Participant characteristics			
*N*	12	12	
Sex			
Male, *n* (%)	5 (46%)	6 (50%)	0.682
Female, *n* (%)	7 (54%)	6 (50%)	
Age (years)	37.9 ± 18.4	39.3 ± 20.9	0.898
BMI (kg/m^2^)	23.2 ± 2.5	22.4 ± 3.3	0.521
Objective sleep measures			
TIB (min)	436.7 ± 39.7	509.8 ± 26.9	< 0.0001
TST (min)	423.5 ± 40.7	505.0 ± 19.2	< 0.0001
SE (%)	97.1 ± 2.0	98.0 ± 1.1	0.214
Haemodynamic measures			
*N*	11	9	
SBP (mmHg)	123.3 ± 15.3	134.6 ± 30.1	0.401
DBP (mmHg)	71.6 ± 10.7	76.9 ± 7.7	0.226
MBP (mmHg)	86.1 ± 15.5	96.2 ± 13.1	0.137
Heart rate			
*N*	12	12	
HR (beats/min)	69.0 ± 8.5	67.4 ± 12.1	0.714

Data presented as mean ± standard deviation unless otherwise specified. Note that four participants including one individual with hypertension had missing BP data due to technical difficulties

*PSQI* Pittsburgh Sleep Quality Index, *ESS* Epworth Sleepiness Scale, *TIB* time in bed, *TST* total sleep time, *SE* sleep efficiency, *SBP* systolic blood pressure, *DBP* diastolic blood pressure, *MBP* mean blood pressure, *HR* heart rate

^a^As multiple participants self-reported a TST equal to the median of 7.5 h, shorter sleep was classified as < 7.5 h and longer sleep classified as ≥ 7.5 h; this explains the slightly higher sample size in the longer versus shorter sleep duration group

**Fig. 2:**
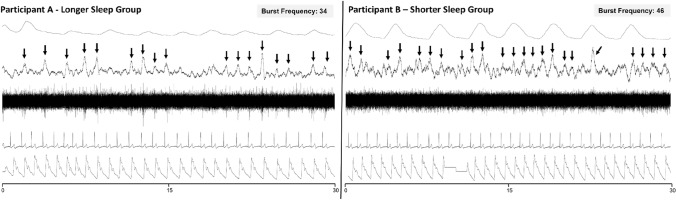
MSNA recordings in participants with shorter versus longer sleep duration. A 30-s MSNA recording from an individual categorised in the **A** longer sleep duration and **B** shorter sleep duration group in a resting awake state showing sequentially from the top: respiration, root mean square waveform of the nerve signal, raw nerve signal, electrocardiogram, and blood pressure. A higher incidence of sympathetic bursts (arrows) was present in participant B (overall burst frequency: 46 bursts/min) compared to participant A (overall burst frequency: 34 bursts/min)

**Fig. 3:**
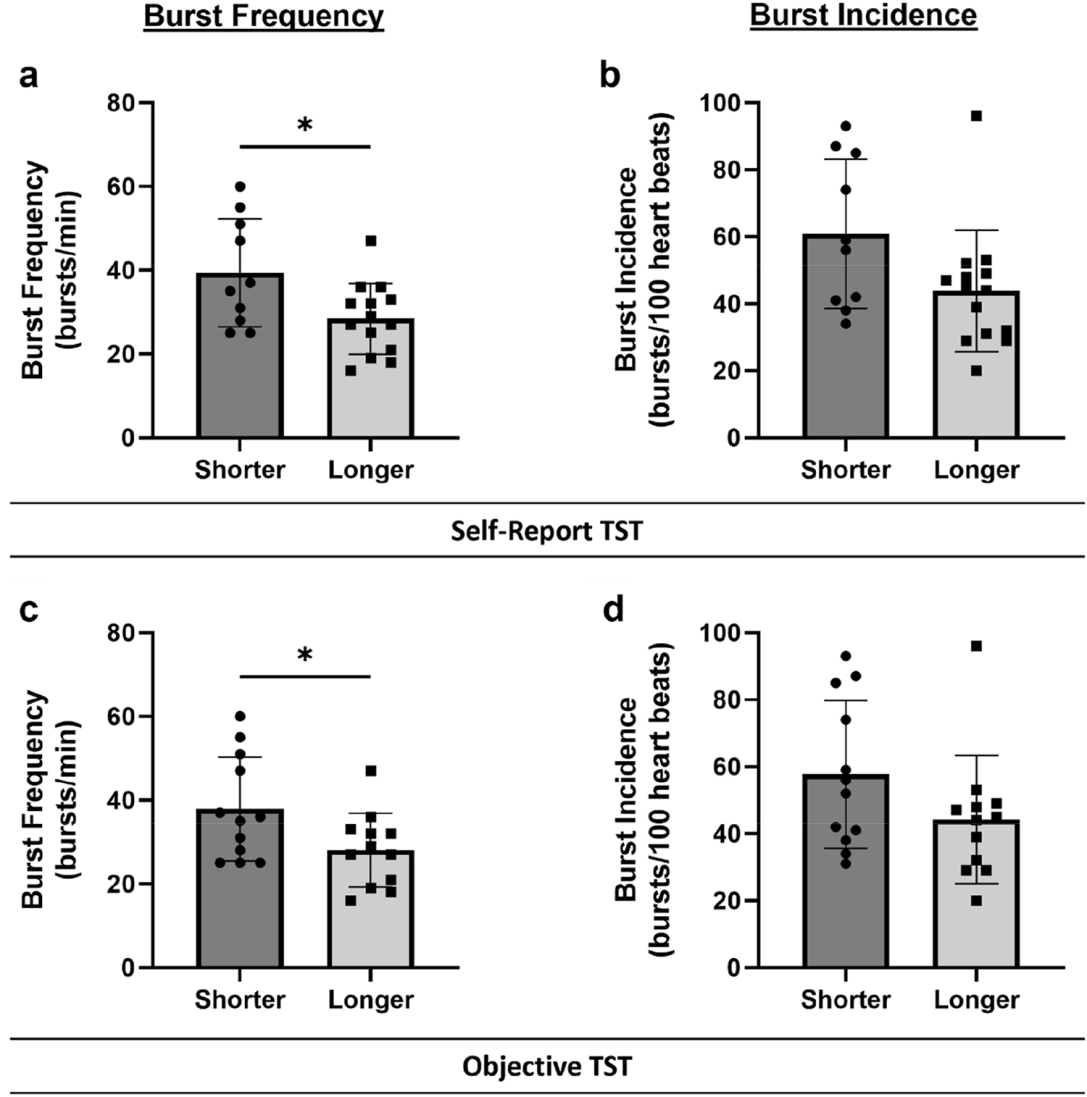
Effect of sleep duration on MSNA. MSNA burst frequency (bursts/min) and burst incidence (bursts/100 heartbeats) in shorter and longer sleep duration groups assessed by (**a** and **b**) self-report total sleep time (TST, min; *n* = 10 shorter sleep duration, *n* = 14 longer sleep duration) and (c and d) objective TST (*n* = 12 per group). Error bars depict standard deviation *p < 0.05

Figure 2 presents an example of MSNA recorded in the resting awake state of an individual in the shorter sleep duration group versus a matched participant with a longer sleep duration. Note the higher number of sympathetic bursts in the short sleeper compared to the longer sleeper (BF: 46 bursts/min vs 34 bursts/min). Group comparisons for MSNA are presented in Fig. 3. Overall, shorter sleepers had significantly higher BF than longer sleepers for both self-report (39.4 ± 12.9 bursts/min vs 28.4 ± 8.5 bursts/min, *p* = 0.019) and objective sleep duration (37.9 ± 12.4 bursts/min vs 28.1 ± 8.8 bursts/min, *p* = 0.036; Fig. 3A and C). However, no significant differences were observed for BI between shorter and longer sleepers for either self-report (60.9 ± 22.3 bursts/100 heartbeats vs 43.9 ± 18.1 bursts/100 heartbeats, *p* = 0.082) or objective sleep duration (57.7 ± 22.0 bursts/100 heartbeats vs 44.3 ± 19.1 bursts/100 heartbeats, *p* = 0.156; Fig. 3B and D).

### Sensitivity analysis

To determine whether hypertensive status affected results, we performed group comparisons where individuals with hypertension were excluded from analyses. There were no differences between groups for BP (SBP, DBP, and MBP) or HR for subjective or objective sleep TST. Both BF and BI were significantly higher in shorter sleepers for self-report (BF: 38.1 ± 13.0 vs 26.8 ± 6.3 bursts/min, *p* = 0.032; BI: 57.3 ± 20.5 vs 39.6 ± 10.5 bursts/100 heartbeats, *p* = 0.034; Figure S2) and objective TST (BF: 37.9 ± 12.3 vs 25.6 ± 5.6 bursts/min, *p* = 0.019; BI: 56.8 ± 19.4 vs 38.0 ± 10.1 bursts/100 heartbeats, *p* = 0.025) compared to longer sleepers. Results were also similar when two participants who reported an ESS > 10 and PSQI > 5 were excluded from the group comparisons (refer to supplement for results).

## Discussion

Epidemiological studies consistently show that sleep duration is associated with an elevated risk of hypertension and CVD [3, 25]. It has been suggested that sympathetic overactivity may underlie the link between shorter sleep duration and hypertension. However, studies have produced conflicting results and have largely been limited to experimental sleep studies rather than real-world sleeping conditions. Utilising both subjective and in-home objective measures of sleep, this study identified that individuals with shorter sleep duration had higher levels of MSNA.

Importantly, this study assessed objective measures via actigraphy, performed in the home setting, to obtain sleep patterns that better reflected participants’ usual routines. Furthermore, subjective measures were assessed, and although subject to recall bias, mirror those of large cohort CVD studies which have mostly relied on self-report measures [26, 27]. Previous studies have shown that subjective reporting of sleep duration either over- or underestimated objective measures [28]. Our results were consistent with these findings, with a tendency to under-report sleep duration (~ 30 min less) compared to objective measures, highlighting the importance of utilising objective sleep measures and the potential differences in interpreting studies that only present self-report sleep data.

Using both subjective and objective measures of sleep, this study demonstrated that shorter sleep duration was associated with higher sympathetic activity. Furthermore, higher levels of daytime sleepiness (assessed by ESS) were associated with elevated BI. Following adjustment for age, a reduction in objective sleep duration by 1 h was associated with an increase in BF of 5.4 bursts/min. When data were split into shorter versus longer sleep duration, BF was 9.8–11 bursts/min higher in individuals with short sleep. Crucially, self-report sleep quality via PSQI and objective sleep quality via SE was not associated with MSNA, suggesting that in this study, elevated MSNA appears to be related to shorter sleep duration per se, rather than sleep disruption. This is not surprising given that our study design aimed to exclude individuals with fragmented sleep (that is, participants with sleep-related pathologies characterised by sleep disruption, such as OSA or insomnia).

Our results support findings from previous studies that show elevated sympathetic activity in experimental sleep-restricted conditions [8, 10, 29]. Although no significant differences in BI were observed, it is important to note that changes in MSNA can be observed when expressed as either measure [30]. In addition, BF provides a similar indication of sympathetic activity as BI in the absence of HR differences between groups, and—given that a change in BF is a measure of the overall change in sympathetic outflow over time—can be seen to reflect central sympathetic outflow more closely.

In this study, although hypertension is a condition associated with high sympathetic activity [23], we chose to include individuals with hypertension, as MSNA appears to remain elevated despite antihypertensive treatment [31]. However, we are aware that the inclusion of participants with hypertension may serve as a confounder in this sample. Importantly, when individuals with hypertension were excluded from the analysis, BF remained significantly higher in the shorter sleep duration group. Furthermore, a significant difference in BI emerged between groups, and the overall group difference in MSNA increased by 2.2 bursts/min (BF) and 5.4 bursts/100 heartbeats (BI) for objective sleep duration.

Overall, the mechanisms underlying the association between short sleep duration and sympathetic overactivity remain unclear. Though MSNA is known to increase with age, it is unlikely that our observations were age-driven, as our cohort was relatively young, and significant associations remained following adjustment for age. An increase in MSNA may arise from alteration in both hormonal and inflammatory processes in those with short sleep duration. Elevations in proinflammatory cytokine secretion have been observed following short sleep duration, with a 4.5% increase in interleukin-6 concentration per hour of sleep loss [32]. Furthermore, one study showed a positive association between interleukin-6 levels and MSNA in a combined healthy and obese sample of similar baseline MSNA [33]. Short sleep duration is also associated with elevated ghrelin, a hormone that innervates the hypothalamus, a structure thought to be involved in modulating sympathetic activity and BP [34, 35]. Ghrelin and MSNA studies are sparse; however, one study observed elevated MSNA post-ghrelin infusion [36]. Intriguingly, sleep regulatory systems are also closely coupled with high-order autonomic centres in the brain involved in cardiovascular modulation, such as the hypothalamus and brainstem [37]. There is limited evidence that this dynamic coupling directly produces sleep-associated MSNA changes. Nonetheless, the hypothalamic paraventricular nucleus, a structure involved in endocrine regulation and stress responses, has been found to influence MSNA during sleep [38]. Moreover, preliminary studies observed elevated activity in the dorsomedial and ventromedial hypothalamus in individuals with high MSNA [39]. Future studies may seek to delineate the specific sleep-autonomic pathways involved.

It is well established that short sleep duration is associated with elevated BP [40, 41]. In contradiction to these epidemiological studies, we observed either lower or no differences in BP in shorter versus longer sleep duration groups. This finding was not surprising, as our study was not powered to assess differences in BP. Furthermore, these differences may have arisen due to the inclusion of treated hypertensive individuals, with more treated hypertensives in the long sleep group. When hypertensive individuals were excluded from analyses, the difference in BP between groups reduced and comparisons remained non-significant. Interestingly, cohort studies with a much larger sample show that a U-shaped or reverse J-shaped relationship exists between sleep duration and hypertension [42]. Hence, it is reasonable to suspect that both very short and very long sleepers might have elevated sympathetic activity. Nevertheless, it is important to note that hypertension observed in those with very long sleep duration is believed to be driven by a confounding factor of poorer health status such as chronic disease, and therefore elevated BP in long sleepers may work via other mechanisms, rather than sympathetic overactivity [42].

Consistent with previous studies, we found no associations between BP with MSNA at rest [43]. Crucially, there is strong evidence suggesting that high sympathetic activation precedes elevated BP, which increases the risk of CVD and hypertension [44, 45]. It is well established that the association between BP and MSNA exists only in older and not younger individuals [46]. Alternatively, it is possible that a resetting of the baroreflex set-point maintains normotension in individuals with short sleep duration observed with sleep deprivation [7, 12]. However, unlike those studies, shorter sleepers did not have elevated DBP or decreased MSNA, which typically indicates a resetting response. Another possibility may be that BP changes in shorter sleepers manifest as blunted nocturnal dipping while daytime BP remains relatively unchanged [47]. Hence, it may be beneficial to conduct 24-h ambulatory BP monitoring to assess both day and night BP changes.

### Limitations

Without the use of polysomnography, we were unable to objectively screen for sleep disorders such as OSA, a condition associated with elevated sympathetic activity [48]. Future studies will benefit from utilising polysomnography, complemented with validated questionnaires and detailed medical histories to exclude potential sleep disorders. Nevertheless, we excluded participants with previously diagnosed OSA or respiratory conditions. We also acknowledge that the use of hip rather than wrist actigraphy may be less accurate and may overestimate measures such as SE [19]. However, this overestimation would be consistent across all participants and would not prevent identification of differences between groups.

Though large cohort studies have typically defined short sleep duration as < 7 h, normal sleep as 7–9 h and long sleep as > 9 h, stratification based on these groupings was not possible due to the small sample size [40, 49]. We do acknowledge that future studies with a larger sample size should examine more granular groupings of sleep duration, including those with both very short and very long sleep duration, given the U-shaped association between sleep duration and BP [6]. MSNA also varies with age, BMI, sex, and hypertensive status [20]. Additionally, sleep-related architecture and circadian processes may differ depending on age [50]. Although we adjusted and performed sensitivity analysis for these confounders, we were unable to delineate differences between factors. Future studies with a larger sample may address these differences. Importantly, this study was cross-sectional and not longitudinal in nature. Therefore, our findings do not imply a *causal* effect of short sleep duration on higher sympathetic activity, but rather an association.

### Perspectives

Sympathetic overactivity is a well-known mechanism driving elevated BP and hypertension. Importantly, sleep duration is modifiable. As such, behavioural intervention to normalise sleep duration in those who experience chronic short sleep may lend itself as a promising candidate for randomised controlled trials to prevent or manage hypertension and CVD. Furthermore, studies targeting sleep neurocircuitry coupled with sympathetic BP modulation may represent potential targets for future studies to understand the pathology of centrally mediated hypertension.

### Conclusion

In conclusion, shorter compared to longer sleep duration is associated with elevated sympathetic activity. Sympathetic overactivity may serve as a precursor to elevated BP, predisposing these individuals to a greater risk of developing hypertension, CVD, and a range of adverse cardiometabolic profiles.

### Supplementary Information

Below is the link to the electronic supplementary material.

Supplementary file1 (DOCX 621 KB)

## Data Availability

Not applicable.

## Abbreviations

MSNA: Muscle sympathetic nerve activity
BF: Burst frequency
BI: Burst incidence
OSA: Obstructive sleep apnoea
BP: Blood pressure
HR: Heart rate
CVD: Cardiovascular disease
BMI: Body mass index
PSQI: Pittsburgh Sleep Quality Index
ESS: Epworth sleepiness scale
TST: Total sleep time
TIB: Time in bed
SE: Sleep efficiency
SBP: Systolic blood pressure
DBP: Diastolic blood pressure
MBP: Mean blood pressure
