# Supplementary material for: The association between sleep duration and muscle sympathetic nerve activity
Source: Clin Auton Res. 2023 Aug 6;33(6):647–57. doi: 10.1007/s10286-023-00965-7 (PMC10751264; doi:10.1007/s10286-023-00965-7)

# **SUPPLEMENTAL MATERIAL**

**Correspondence**

Dr Stephanie Yiallourou

Turner Institute for Brain and Mental Health, Monash University, Melbourne, Australia

Email: [stephanie.yiallourou@monash.edu](mailto:stephanie.yiallourou@monash.edu)

**SUPPLEMENTAL METHODS**

*Sleep questionnaires*

Two self-administered sleep questionnaires were used to assess self-report sleep quantity, quality, and potential sleep disorders. The Pittsburgh Sleep Quality Index (PSQI) is a 19-item questionnaire used to assess sleep quality in the past month, with seven components assessing: sleep duration, latency, quality, efficiency, disturbances, sleep medications, and sleep-driven daytime dysfunction [1]. A global score of >5 is defined as clinically significant poor sleep quality. The Epworth Sleepiness Scale (ESS) was utilised to assess daytime sleepiness, an eight-item questionnaire that rates sleepiness in a variety of daytime scenarios, with scores >10 considered clinically significant [2]. Both questionnaires were scored in adherence to established instructions [2, 3].

*Actigraphy*

Objective sleep was assessed using 24-hour, 7-day/night actigraphy (GT3X-BT, ActiGraph, FL, USA). The device was fitted on participants’ hips along their mid-axillary line. However, hip placement was chosen, as this study was part of a larger-scale study, which was also interested in physical activity data throughout the day as well as nighttime sleep. Hip actigraphy is considered more reliable for physical activity measures [4], though is less accurate for sleep assessment compared to wrist actigraphy when compared against gold-standard polysomnography [5]. Using the Cole-Kripke algorithm, validations studies of both hip and wrist worn accelerometry against gold-standard polysomnography show moderate agreement (defined a priori as r >0.6 based on spearman rank test) between hip and wrist worn actigraphy with PSG. Importantly, hip-worn accelerometers had the strongest correlation to PSG assessed TST (r=0.73). Sleep diaries often used with actigraphy were provided for logging in and out of bedtimes and periods of non-wear [6]. Actigraphy recordings were analysed by the same trained researcher with specialised software (Actilife 6.8.1, ActigraphTM) in 60-second epochs, using a standardised protocol. Activity data and worn times were manually inspected and validated against diary entries, followed by determining sleep and awake states based on activity using the validated Cole-Kripke algorithm [5].

Days with missing activity or recordings comprising less than four days were excluded, potentially due to device error or non-wear, to minimise misrepresentation of participants’ normal sleep patterns. Total sleep time (TST, min), time in bed (TIB, min – derived from in and out of bedtimes) and sleep efficiency (SE, % – derived from TST divided by TIB) were averaged across the 7 nights of the recording.

*Microneurography and Physiological Parameters*

Microneurography was performed via previously established protocols [7]. Participants were seated in a semi-recumbent position with their right leg extended and supported horizontally. The common peroneal nerve was located using palpitation of the fibular head followed by electrical stimulation with a probe. Stimulation evoked visible muscle twitches in the foot at varying intensities. An optimal neural recording site was selected based on the greatest strength of muscle twitch observed with the lowest current needed. A reference tungsten microelectrode (200μm diameter with a three to five μm tip, variable impedance, FHC, Bowdoin, ME, USA) was inserted subdermally nearby followed by percutaneous insertion of the active microelectrode at the recording site. Intraneural stimulation was performed while altering the angle and depth of the electrode until twitches remained observable at a current of 0.02 mA, serving as a recording site for MSNA. Confirmatory tests were performed to ensure successful penetration into a muscle fascicle. Neural activity was amplified (Neuro Amp Ex, ADInstruments, Sydney, Australia) and sampled at 10kHz.

A respiratory belt transducer strapped around the thorax and a three-lead chest electrocardiogram (ADInstruments, Sydney, Australia) was used to measure respiratory movements and electrocardiographic activity, respectively. Continuous BP was recorded at rest using an appropriately sized, non-invasive photoplethysmographic cuff placed around the left ring or middle finger (NOVA, Finapres Medical Systems, Enschede, the Netherlands). Continuous BP measures were calibrated against an oscillometric brachial blood pressure measurement obtained from the non-monitored (right) arm using an upper arm cuff. The height correction unit was zeroed and implemented as per manufacturer’s specifications. Baseline parameters were recorded for 15 minutes to obtain a 5-minute artifact-free recording to be used for calculating baseline parameters.

MSNA was analysed offline using LabChart 7 (ADInstruments, Sydney, Australia). The nerve signal was root mean square processed to easily identify spontaneous MSNA bursts. Bursts were ascertained by identifying negative-going spikes using careful inspection of the nerve signal and close coupling of bursts to ECG waves. Artifacts or brief electrical spikes were deemed false positives and excluded from analyses. Bursts were quantified according to burst frequency (BF, bursts/min) and burst incidence (BI, bursts/100 heartbeats).

BP and HR were calculated via peak detection of both BP and ECG signals, respectively, and analysed offline with Labchart 7. Beat-to-beat measures of systolic blood pressure (SBP, mmHg), diastolic blood pressure (DBP, mmHg), mean blood pressure (MBP, mmHg), and HR (beats/min) were obtained and averaged across 10 to 15 minutes of baseline recordings for each participant. MBP was calculated using the following equation: MBP=DBP+1/3(SBP-DBP) [8]. Individuals were classified as hypertensive according to ≥140/90 mmHg (SBP/DBP) or prescribed hypertensive medication [9].

**REFERENCES**

1. Zhong Q-Y, Gelaye B, Sánchez SE, Williams MA (2015) Psychometric Properties of the Pittsburgh Sleep Quality Index (PSQI) in a Cohort of Peruvian Pregnant Women. J Clin Sleep Med 11:869–877. https://doi.org/10.5664/jcsm.4936

2. Johns MW (1991) A new method for measuring daytime sleepiness: the Epworth sleepiness scale. Sleep 14:540–545. https://doi.org/10.1093/sleep/14.6.540

3. University of Pittsburgh Center for Sleep and Circadian Science (2010) PSQI Scoring

4. Aadland E, Ylvisåker E (2015) Reliability of the Actigraph GT3X+ Accelerometer in Adults under Free-Living Conditions. PLoS One 10:e0134606. https://doi.org/10.1371/journal.pone.0134606

5. Full KM, Kerr J, Grandner MA, et al (2018) Validation of a physical activity accelerometer device worn on the hip and wrist against polysomnography. Sleep Health 4:209–216. https://doi.org/10.1016/j.sleh.2017.12.007

6. Horne RSC, Biggs SN (2013) Actigraphy and Sleep/Wake Diaries. The Oxford Handbook of Infant, Child, and Adolescent Sleep and Behavior. https://doi.org/10.1093/oxfordhb/9780199873630.013.0014

7. Macefield VG (2021) Recording and quantifying sympathetic outflow to muscle and skin in humans: methods, caveats and challenges. Clin Auton Res 31:59–75. https://doi.org/10.1007/s10286-020-00700-6

8. Chaudhry R, Miao JH, Rehman A (2022) Physiology, Cardiovascular. In: StatPearls. StatPearls Publishing, Treasure Island (FL)

9. Oparil S, Acelajado MC, Bakris GL, et al (2018) Hypertension. Nat Rev Dis Primers 4:18014. https://doi.org/10.1038/nrdp.2018.14

**SUPPLEMENTAL RESULTS**

*Sensitivity analysis excluding participants with ESS>10 and PSQI>5*

As this study did not include polysomnography to screen out sleep disorders, we performed a sensitivity analysis, where those with highest risk of a sleep disorder were excluded. Results were similar, when two participants who reported and ESS>10 and PSQI>5 were excluded from the groups comparisons. Overall, shorter sleepers had significantly higher BF compared to longer sleepers for both self-report (38.6 ± 11.1 bursts/min vs 28.4 ± 8.5 bursts/min, p=0.044) and objective sleep duration (37.0 ± 10.7 bursts/min vs 28.1 ± 8.8 bursts/min, p=0.050). However, no significant differences were observed for BI between shorter and longer sleepers for either self-report (60.0 ± 22.2 bursts/100 heartbeats vs 43.9 ± 18.1 bursts/100 heartbeats, p=0.105) or objective sleep duration (56.3 ± 21.6 bursts/100 heartbeats vs 44.3 ± 19.1 bursts/100 heartbeats, p=0.187.

**FIGURE LEGENDS**

**Fig. S1 Correlation between Objective and Self-Report Sleep Duration**

The correlation between objective total sleep time (TST, min) and self-report TST (min) is shown (n=24) *P<0.0001.

**Fig. S2** **Comparison of MSNA between shorter and longer sleepers, excluding hypertensives, grouped by subjective and objective sleep duration**

MSNA parameters (burst frequency, bursts/min; burst incidence, bursts/100 heartbeats) were compared between groups, excluding individuals with hypertension, according to groupings for (a and b) self-report total sleep time [TST, min; shorter (n=9), longer (n=9)] and (c and d) objective TST, [shorter (n=10), longer (n=8)]. Error bars depict standard deviation *P<0.05

**Fig. S3 Correlation between Age, BMI, and MSNA**

The correlation between MSNA burst frequency (bursts/min) and burst incidence (bursts/100 heartbeats) with (a and b) age (years) and (c and d) body mass index (kg/m^2^) (n=24) *P<0.05

**Fig. S1**

**
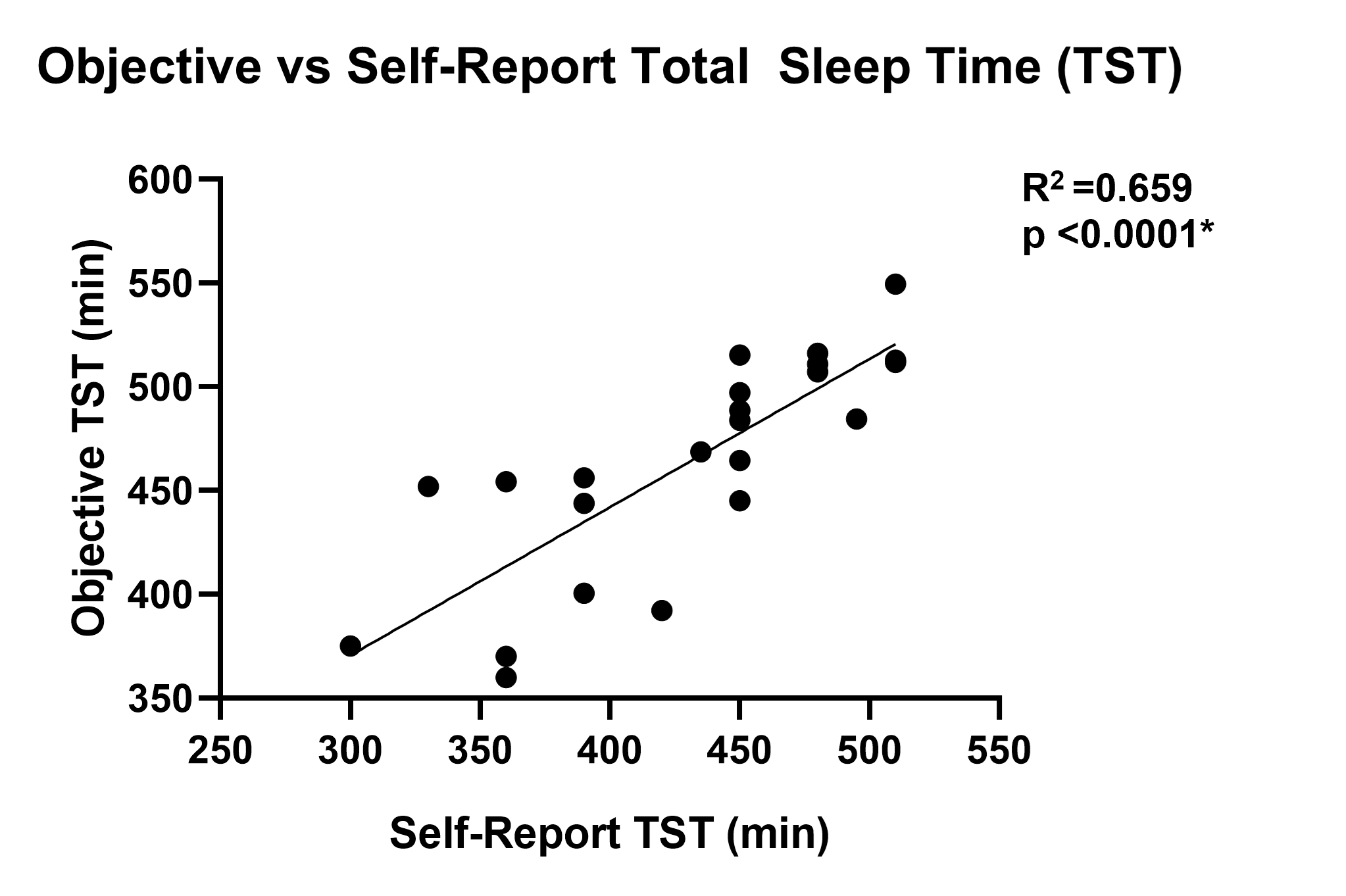
**

**Fig. S2**


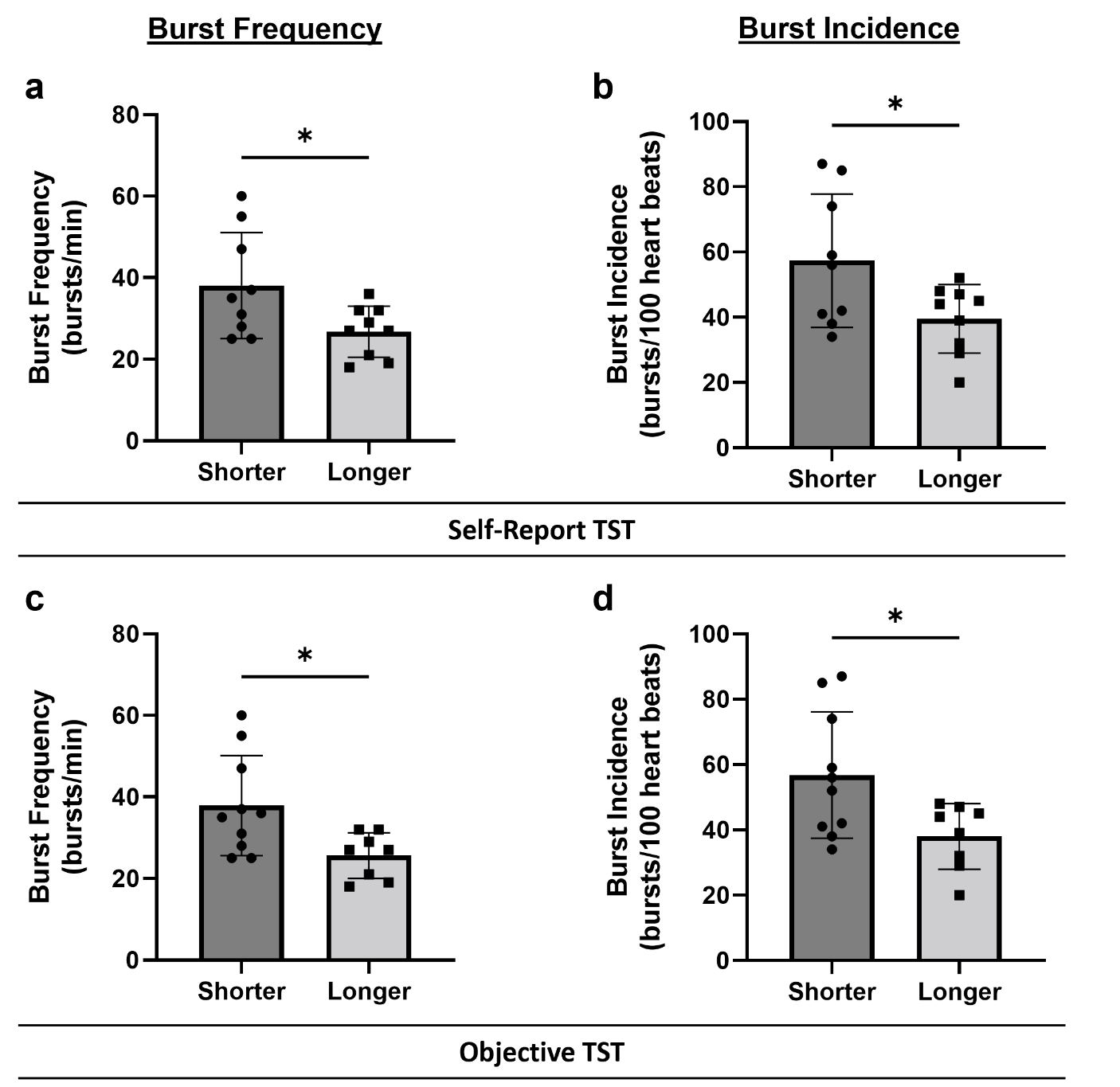


**Fig. S3**


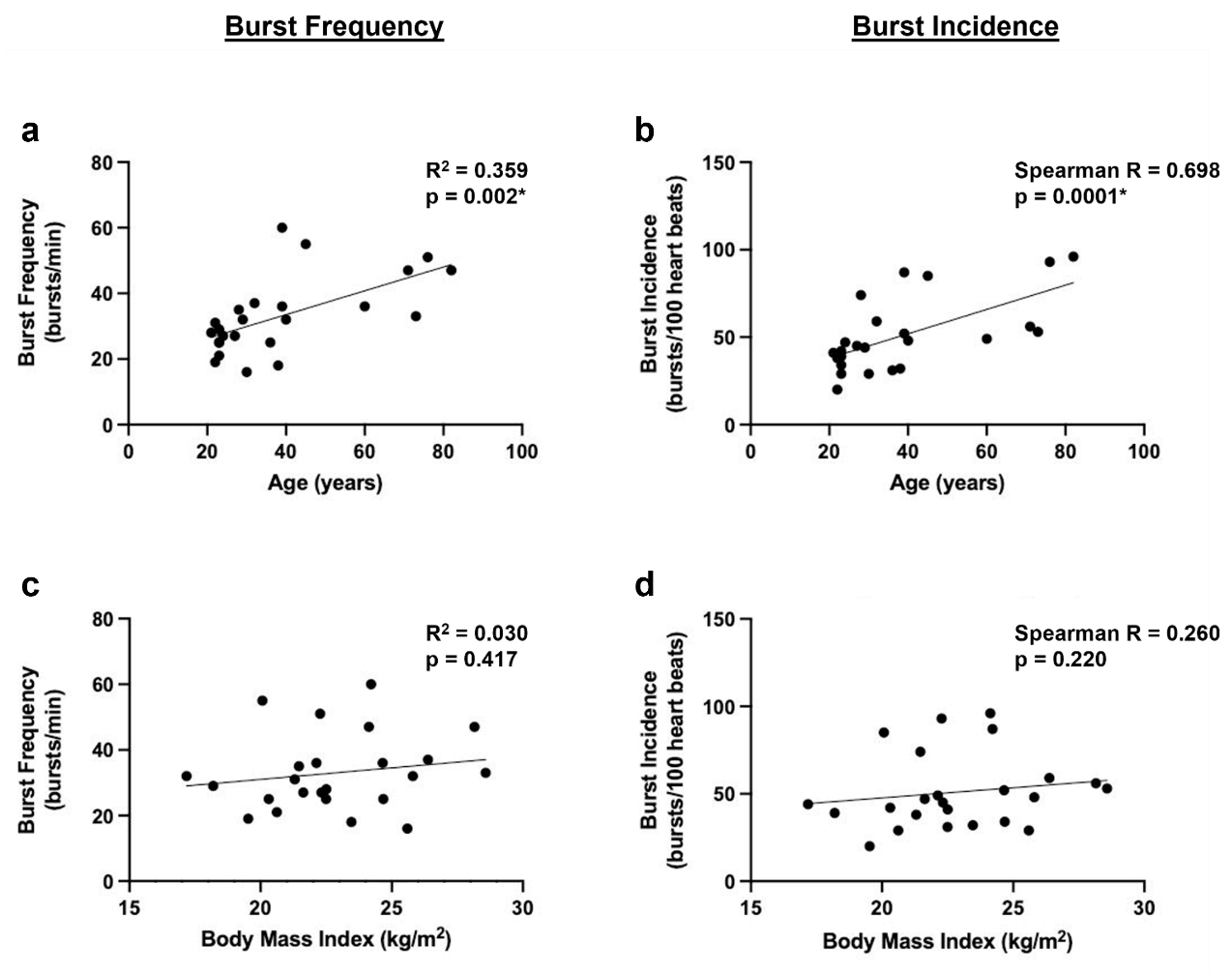


**EPWORTH SLEEPINESS SCALE**


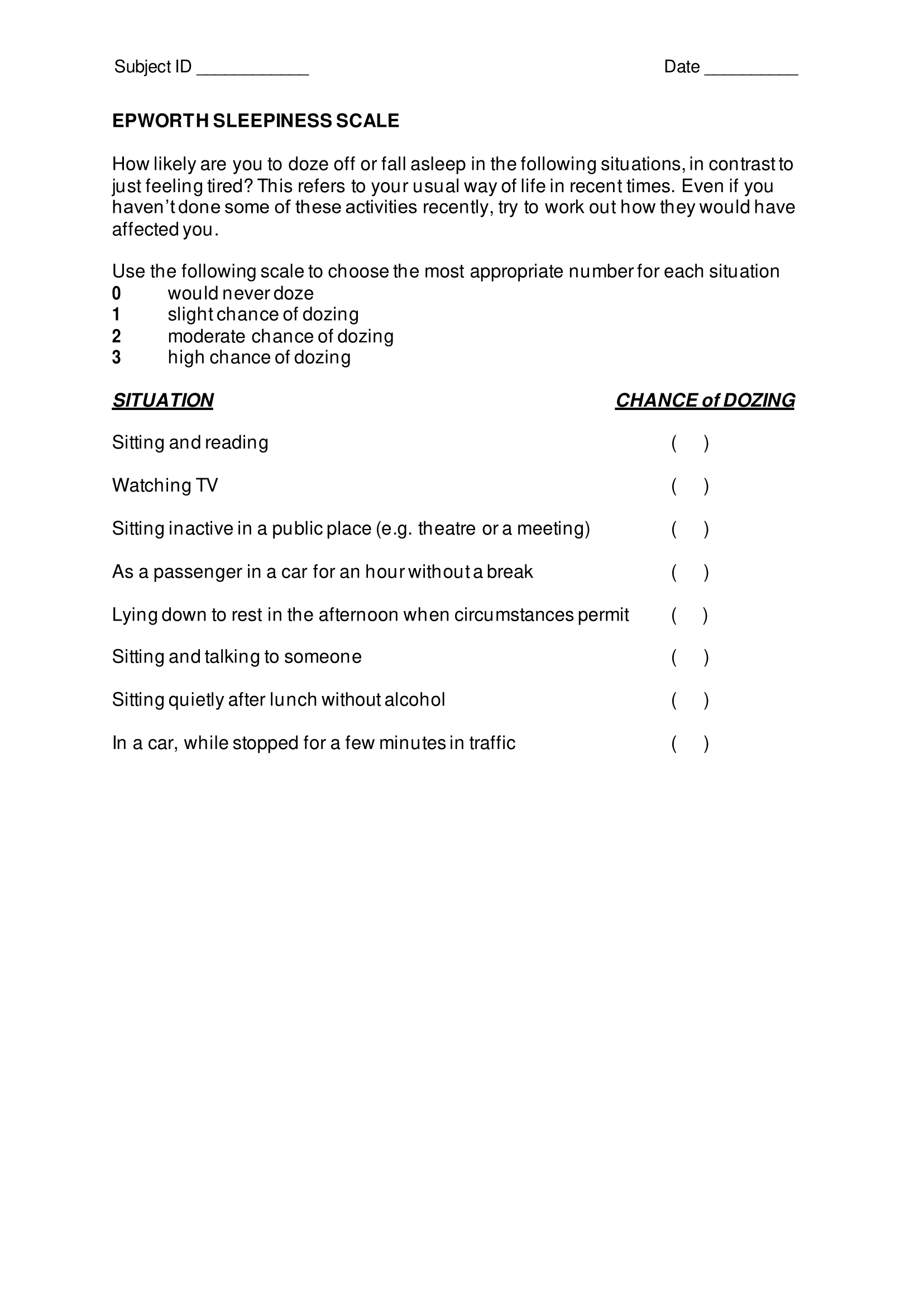


**PITTSBURGH SLEEP QUALITY INDEX**


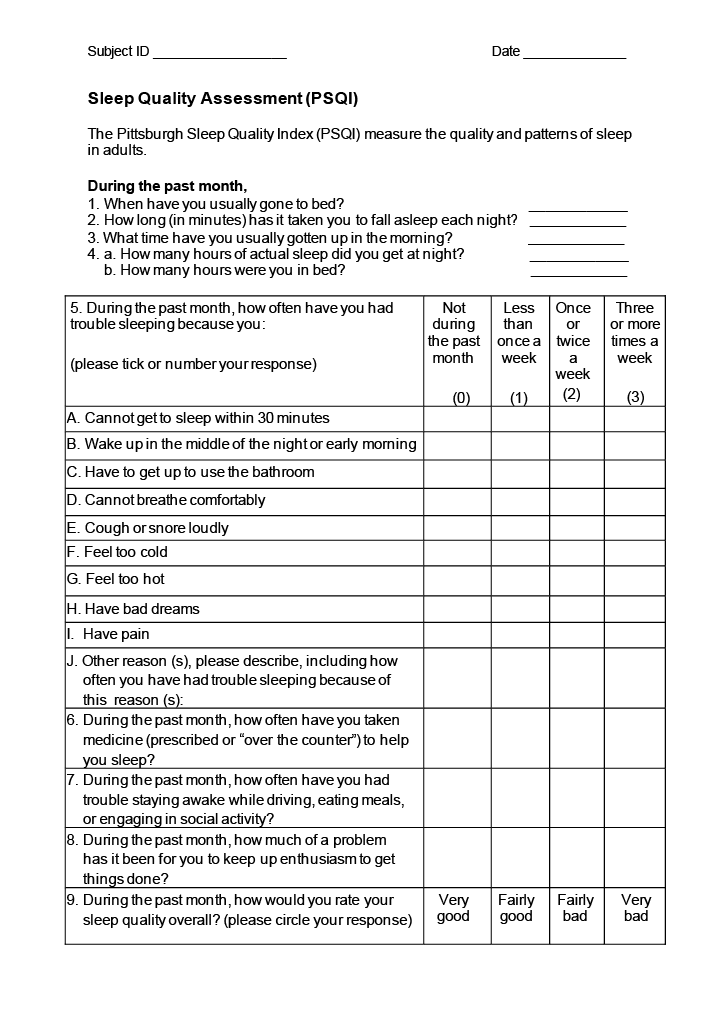

Supplement: Supplementary file 1 — Supplementary file1 (DOCX 621 KB) [file 10286_2023_965_MOESM1_ESM.docx]
